# The Pneumoconiosis Renaissance: Revisiting the Pulmonary Pathology of Poorly Soluble Low Toxicity Particles: Insights from Rodent Inhalation Studies on Titanium Dioxide Nanoparticles

**DOI:** 10.3390/nano16040230

**Published:** 2026-02-11

**Authors:** Shotaro Yamano, Dirk Schaudien, Yumi Umeda

**Affiliations:** 1 National Institute of Occupational Safety and Health, Japan Organization of Occupational Health and Safety, 2-26-1 Muraoka-Higashi, Fujisawa 251-0015, Kanagawa, Japan; 2 Fraunhofer Institute for Toxicology and Experimental Medicine ITEM, 30625 Hannover, Germany

**Keywords:** poorly soluble low toxicity particles (PSLTs), titanium dioxide nanoparticles (TiO_2_ NPs), pneumoconiosis, spatial resolution, biological mask, pulmonary dust foci (PDF), dust macule, rat overload, adverse outcome pathway (AOP), risk assessment, interspecies differences, comparative pathology

## Abstract

Historically, the toxicological evaluation of poorly soluble low toxicity particles (PSLTs), such as titanium dioxide nanoparticles (TiO_2_ NPs), distinct from conventional pigment-grade TiO_2_, has focused on carcinogenicity and lung overload, leaving their pathological function in the development of pneumoconiosis undefined. In this study, we initiated a “Pneumoconiosis Renaissance”, redefining the human “Gold Standard” of pneumoconiosis pathology as a primarily interstitial “Dust Macule (DM) to Mixed Dust Fibrosis (MDF) axis”. In contrast, rats developed a species-specific “Airspace-Dominant Phenotype” (Pulmonary Dust Foci) driven by airspace stagnation. Integrating recent continuous inhalation exposure and recovery after inhalation exposure studies, we demonstrate that this overwhelming alveolar pathology in rats acts as a “Biological Mask”, physically superimposing upon and obscuring human-relevant interstitial sequestration. Crucially, however, extended recovery periods can unmask these interstitial events, revealing the true underlying pathology. We propose that future risk assessments and Adverse Outcome Pathways (AOPs) must incorporate spatial resolution. By rigorously segregating sensitive rat-specific airspace events from human-relevant interstitial remodeling, we can accurately bridge the interspecies gap. This review argues that rather than discarding the rat model, we must learn to decode it—using spatial distinctions to filter the airspace mask and evaluate the true interstitial risk of inhaled biodurable particles.

## 1. Introduction

### 1.1. Pneumoconiosis: Historical Background

Pneumoconiosis, is defined as an occupational lung disease characterized primarily by fibrotic changes in the lungs caused by the inhalation of dusts [1]. It is one of the oldest recorded occupational diseases, with historical records dating back to around 400 BC, when Hippocrates (460–370 BC), known as the Father of Medicine, described respiratory difficulties in metal diggers [2]. Subsequently, the history of occupational medicine was first systematically described in the 16th century by Paracelsus (1493–1541) and Georgius Agricola (1494–1555), who focused particularly on health issues among workers in manufacturing plants and mines [2]. With the development of underground coal mining, pneumoconiosis began to be associated with coal mining [3,4,5].

In the 19th century, Rudolf Virchow (1821–1902), the father of modern pathology, established the principles of cellular pathology [6], which laid the groundwork for understanding the tissue reaction to dust. Although early interpretations of pigmentation in miners’ lungs were sometimes confounded with endogenous causes [7] (also see the Introduction by Donaldson et al. [8]), the establishment of histopathology allowed for the recognition of exogenous dust accumulation as the primary cause of melanosis of the lung. Today, pneumoconiosis remains a critical global health issue, and research is continuing with the fundamental pathological criteria derived from human cases serving as the essential baseline for toxicological investigations.

### 1.2. The Gold Standard of Human Pathology: Insights from Whole Lung Sections

While the association between dust and disease has been recognized by leading experts for hundreds of years (as noted above) and has been generally accepted since the mid-1990s [5], understanding the precise distribution and severity of pneumoconiosis was long hindered by the limitations of standard histological techniques, which typically examine only small tissue blocks. A revolution in respiratory pathology occurred in 1949 with the development of the Gough–Wentworth whole lung section technique by Jethro Gough and James E. Wentworth at the Welsh National School of Medicine [9]. This technique allowed for the preservation of the entire lung as a single, paper-mounted thin slice, enabling pathologists to visualize the disease as a macroscopic map. As reviewed by Donaldson et al., this technique established the Gold Standard for correlating radiological findings with pathological reality, revealing features like emphysema and spotty dust distribution that were invisible on standard X-rays [10].

**Figure 1:**
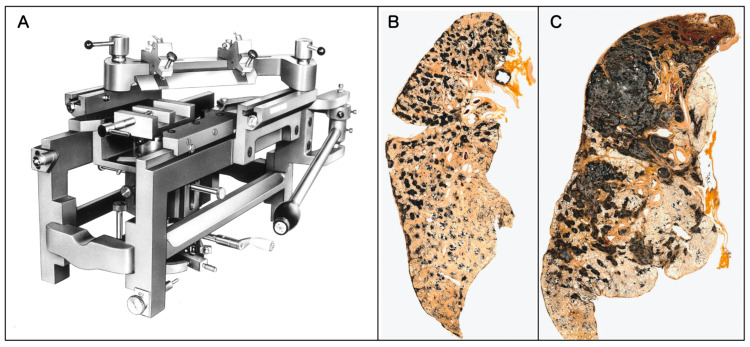
The “Gold Standard” of pneumoconiosis pathology: methodology and macroscopic topography. (**A**) Large Universal Sliding Microtome (US-111, Yamato Kohki Industrial Co., Ltd., Saitama, Japan). This specialized equipment is essential for preparing Gough–Wentworth whole lung sections, allowing for the visualization of the entire lung architecture on a single plane. (**B**) Macroscopic whole lung section of Mixed Dust Pneumoconiosis. Case of a 63-year-old male with 34 years of exposure to dusts as a tunnel construction worker and 3 years of exposure to dusts as a coal miner. The section revealed numerous dust macules and Mixed Dust Fibrosis (MDF) nodules accumulating predominantly in the upper lobes (Japanese Classification of Pneumoconiosis: Type 3/3 q). (**C**) Macroscopic whole lung section of Silicosis with PMF. Case of a 65-year-old male stone mason (47 years of exposure). The lesion had progressed to Progressive Massive Fibrosis (PMF), characterized by large, coalescent black masses destroying the upper lobe architecture (Japanese Classification of Pneumoconiosis: Type 4C). Reprinted with permission from Ref. [11]. Copyright 2008, Japan Labor Health and Welfare Organization (currently JOHAS).

In this review, to illustrate the hallmark features of human pneumoconiosis, we present original Gough–Wentworth whole lung sections from the archives of Hokkaido Central Rosai Hospital, Japan (Figure 1). Hokkaido was a major center of the coal mining industry in Japan, and this hospital has historically played a central role in the diagnosis and treatment of pneumoconiosis.

Differences in bronchial branching angles and lymphatic clearance efficiency affect the accumulation and retention of dust in the lung and the development of pneumoconiosis [12,13]. The initial development of pneumoconiosis, indicated by the presence of small pneumoconiotic opacity patterns in chest radiographs, has been reported. Amandus et al. (1974) examined 627 radiographs of U.S. coal miners and reported the presence of small opacities in the right and left upper, middle, and lower zones of the lung [14]. Young et al. (1992) examined the chest radiographs of 98 former U.S. coal miners who were diagnosed with coal miner’s pneumoconiosis [15]. They reported that most small rounded opacities and most irregular opacities occurred in the middle and lower lung zones. Laney and Petsonk (2012) examined chest radiographs of 2467 U.S. coal miners [16]. They reported that 30.7% of small pneumoconiotic opacities occurred in the upper zone of the lung, 37.1% in the middle zone, and 32.1% in the lower zone. However, they found that upper zone involvement was more common when the primary shape of the opacity was round, and that lower zone involvement was more common when the primary shape of the opacity was irregular. Rehman et al. (2022) characterized the patterns of small opacities in chest radiographs of 330 New Mexico coal miners and found that almost all of these pneumoconiotic opacities were irregular in shape and over 95% of the opacities were lower zone predominant [17]. In contrast to the development of small opacities, studies that examined miners’ lungs for more advanced cases of pneumoconiosis exhibiting Progressive Massive Fibrosis (PMF), where localized nodules coalesce into larger, irregular masses exceeding 1 or 2 cm in diameter, reported a different pattern of development in the lung. Halldin et al. (2022) examined chest radiographs of 204 coal miners previously identified to have large opacities and 7 chest radiographs of coal miners with indication of coalescence of small opacities [18]. A total of 41% of the large opacites occurred in the upper right lung zone, 28% in the upper left lung zone, and 31% in the middle or lower lung zones. Sari et al. (2022) examined Computed Tomography images obtained from 90 patients with pneumoconiosis and reported that 86 patients had PMF in the upper lobes of the lung and only 4 patients had PMF in the middle or lower lung lobes [19].

The reasons for the discrepancies in the development of initial pneumoconiosis lesions and the development of more advanced lesions are currently unknown. One possibility is that, as noted in Occupational, Environmental and Iatrogenic Lung Disease [13], there is greater respiratory excursion in the lower lung zones, resulting in greater air flow in the lower zones. Consequently, the lower zones would receive more dust than the upper zones, but would also have greater dust clearance activity. Therefore, while dust nodules could develop more readily in the lower lung than in the upper lung, the dust nodules that develop in the upper lung would be less likely to be cleared, allowing for more ready development of advanced pneumoconiosis lesions in the upper lung.

As vividly demonstrated in Figure 1, Gough–Wentworth whole lung sections revealed a critical feature of advanced human pneumoconiosis lesions: the lesions are not diffusely or homogeneously distributed from the apex to the base. Instead, they exhibit a distinct regional heterogeneity. The dust macules (DM) and fibrotic nodules that eventually give rise to advanced lesions, such as the lesion shown in Figure 1, do not form randomly but form in specific hotspots, most notably in the upper lobes, often more pronounced in the right upper lobe. The macroscopic findings from Gough–Wentworth whole lung sections represent the Gold Standard phenotype of human dust exposure and retention. Recognizing this specific topographical pattern is essential when evaluating animal models, as interspecies differences in posture (bipedal vs. quadrupedal) and airway geometry may lead to fundamentally different deposition and retention patterns in rodents.

### 1.3. Comparative Anatomy of the Lung Interstitium: Defining the “Broad” Compartment

Before discussing specific lesions, it is essential to establish a common anatomical framework, as the distribution of interstitial tissue differs significantly between humans and rodents. In pulmonary pathology, the interstitium is conceptually divided into two distinct compartments based on structure and function. The first is the narrow interstitium (alveolar septal interstitium), which supports gas exchange: denoted as septal interstitium in Gil and McNiff and shown as the thin layer of connective tissue surrounding the alveolus in Figure 1 of Taylor et al. [20,21]. The second is the broad interstitium (loose connective tissue), which contains lymphatic vessels and serves as the primary drainage route for particulates inhaled into the alveoli: denoted extraalveolar interstitium in Gil and McNiff and shown as the thicker interstitium in Figure 1 of Taylor et al. This broad compartment encompasses the axial interstitium (also known as the peribronchiolar interstitium or the interstitium of the broncho-vascular bundle [BVB]), interlobular interstitium, and subpleural interstitium.

**Figure 2:**
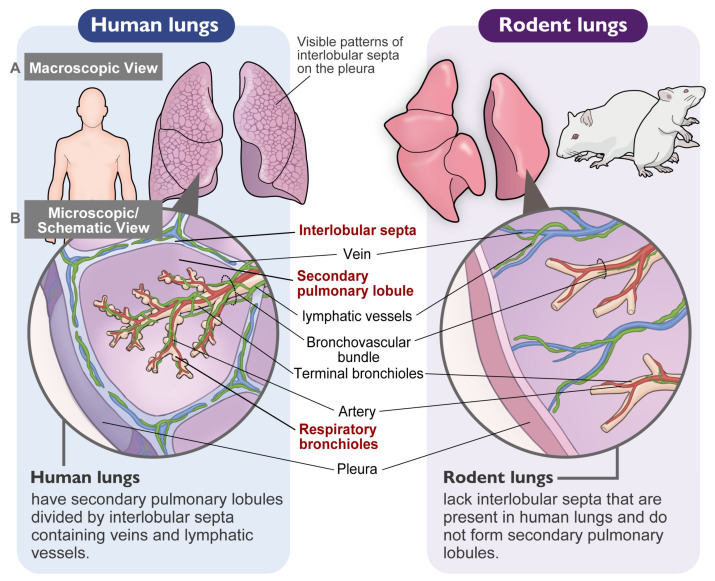
Schematic comparison of pulmonary architecture. The anatomical basis of species differences. (**A**) Macroscopic View: Human lungs (**left**) exhibit a distinct “polygonal pattern” on the visceral pleura, reflecting the boundaries of secondary lobules. In contrast, rat lungs (**right**) show a smooth surface, lacking lobular demarcation. (**B**) Microscopic/Schematic View: This panel illustrates the secondary lobule architecture and the “Broad Interstitium” concept. In humans (**left**), the secondary lobule is a distinct polygonal unit defined by thick connective tissue walls (Interlobular Septa and Subpleural Septa) containing veins and lymphatic vessels. Particles are drained towards the Axial (peribronchiolar) interstitium and Septal (interlobular) interstitium, forming Dust Macules. In rats (**right**), the lung parenchyma is continuous without septal division. However, the Perivenous Interstitium functionally corresponds to the human interlobular septa. Recognizing this “functional equivalence” is crucial: while the rat’s primary response is often alveolar (airspace), the subtle perivenous sequestration represents the true homolog to human interstitial pathology.

A critical challenge in comparative pathology is the lack of well-defined secondary lobules in rodents (Table 1). As elucidated in our recent comparative anatomy study encompassing over 30 mammalian species [22], rodents lack true interlobular septa (Figure 2A,B). However, we demonstrated that the perivenous interstitium in non-lobulated species (such as rodents) functionally and anatomically corresponds to the interlobular septa in lobulated species (such as humans).

Therefore, to bridge the anatomical gap between species, this review adopts the term “Broad Interstitium” to collectively refer to this lymphatic-rich loose connective tissue—specifically the peribronchiolar (axial), perivenous (interlobular equivalent), and subpleural regions. Recognizing that pneumoconiosis is primarily a disease of the Broad Interstitium, while conditions such as Diffuse Alveolar Damage (DAD) and Nonspecific Interstitial Pneumonia (NSIP) primarily affect the Narrow Interstitium, is key to understanding the Dust Macule to Mixed Dust Fibrosis axis (DM–MDF axis), discussed below. However, a frequent source of confusion in particle toxicology is the indiscriminate use of the term “interstitial fibrosis”. Researchers often interpret “alveolar septal fibrosis” (thickening of the Narrow Interstitium)—a typical response in rat lung overload—as a precursor to human pneumoconiosis caused by poorly soluble low toxicity particles (PSLTs). We argue that this is a false equivalence. True pneumoconiosis implies architectural remodeling of the Broad Interstitium and compromising the lymphatic drainage system, not adversely affecting the function of the alveolar septa.

### 1.4. Histopathological Classification: Macules, Nodules, and Fibrosis

The macroscopic lesions observed in whole lung sections are histologically composed of specific microscopic units. Based on the classic pathology standards established by the College of American Pathologists (CAP) and experts such as Kleinerman et al. [23], and further endorsed by modern pulmonary pathology authorities including Butnor and Roggli [24], Mukhopadhyay [25], and Katzenstein [26], pneumoconiotic lesions are generally classified into three fundamental types, discussed below. Furthermore, regarding the specific morphology of lesions induced by lower-toxicity dusts, the criteria for mixed-dust pneumoconiosis (MDP) proposed by Honma et al. [27] provides an essential framework regarding MDP histopathology. Understanding these definitions is crucial for distinguishing simple dust accumulation from pathogenic fibrosis.

**Figure 3:**
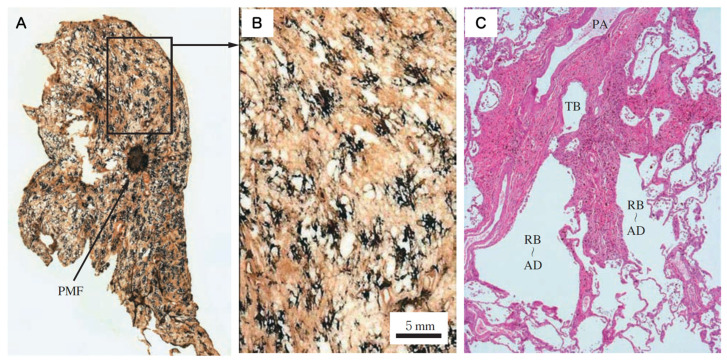
Histopathological characteristics of the Dust Macule (DM), the fundamental lesion of the interstitial pneumoconiosis axis. (**A**) Macroscopic view of a whole lung section (Gough–Wentworth). While a focal Progressive Massive Fibrosis (PMF) lesion is noted in the center, the background lung parenchyma exhibits a widespread distribution of black macules. The rectangular frame indicates the area magnified in (**B**). (**B**) Close-up view of the macules. These lesions appear as non-palpable, ill-defined black spots (extending slightly over 5 mm), distinctly associated with focal emphysema (centrilobular emphysema in [10]). (**C**) Medium-power photomicrograph of a dust macule. The lesion is characterized by fibrous thickening of the interstitium surrounding the terminal bronchiole (TB), respiratory bronchiole (RB), and alveolar duct (AD), along with the accompanying pulmonary artery (PA). Note the dilation of the bronchiolar and alveolar lumens (focal emphysema), illustrating the architectural remodeling of the “Broad Interstitium”. Reprinted with permission from Ref. [11]. Copyright 2008, Japan Labor Health and Welfare Organization (currently JOHAS).

**Figure 4:**
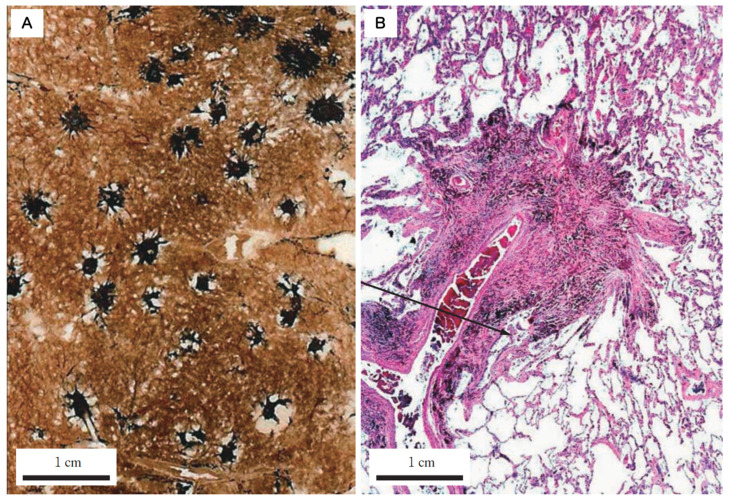
Histopathological characteristics of the Mixed Dust Fibrosis (MDF) nodule. (**A**) Macroscopic close-up view of a whole lung section. The image reveals multiple stellate-shaped black nodules, measuring several millimeters in diameter, with surrounding traction emphysema caused by fibrous contraction. (**B**) Low-power photomicrograph. The nodule exhibits a distinct stellate architecture, with fibrosis containing heavy dust deposition extending irregularly along the distal axial interstitium into the alveolar duct region. The lesion is situated adjacent to the terminal bronchiole (TB) and pulmonary artery (PA), confirming its localization within the axial interstitium. Unlike classic silicotic nodules, these lesions are rich in cellular components (histiocytes/macrophages and fibroblasts) and lack concentric hyalinized whorls. Reprinted with permission from Ref. [11]. Copyright 2008, Japan Labor Health and Welfare Organization (currently JOHAS).

1. **Dust Macule (DM)**: A non-palpable collection of dust-laden macrophages accumulating primarily in the peribronchiolar interstitium. In coal worker’s pneumoconiosis (CWP), this is often associated with the destruction of adjacent alveolar septa, termed focal emphysema. Donaldson et al. emphasized that the macule is characterized by reticulin fibers rather than collagen and is distinctively linked to focal dust emphysema [10]. Importantly, the macule itself exhibits minimal to no collagen deposition (Figure 3).
2. **Nodule (Fibrotic)**: A palpable, discrete lesion characterized by collagen deposition. It is critical to distinguish between two subtypes of nodules based on the nature of the dust. The classic Silicotic Nodule features concentric, whorled hyalinized collagen with a sharp border, typical of high-silica exposure. In contrast, dusts with lower silica content or poorly soluble low toxicity particles (PSLTs) typically induce the Mixed Dust Fibrotic (MDF) nodule (also known as the mixed dust pneumoconiosis nodule). As defined by Honma et al. [27], the MDF nodule is distinctively characterized by a stellate (star-shaped) or irregular distribution of collagen fibers extending into the surrounding interstitium, intermingled with abundant dust-laden macrophages (Figure 4).

3. **Progressive Massive Fibrosis (PMF)**: A large area of confluent fibrosis, typically defined as a lesion greater than 1 cm in diameter (based on ILO classification), resulting from the aggregation of nodules and often associated with vascular destruction.

This classification system—distinguishing between the simple accumulation of macrophages (Macules) and the development of irreversible collagenous structures (Nodules/PMF)—is the most critical metric for evaluating the toxicity of inhaled particles.

### 1.5. Poorly Soluble Low Toxicity Particles (PSLTS)/Poorly Soluble Particles (PSPS) and TiO_2_: The “Dm–Mdf Axis”

Particles characterized by low solubility and low inherent cytotoxicity have historically been categorized as “Poorly Soluble Low Toxicity Particles (PSLTs)”. Titanium dioxide (TiO_2_), extensively used as a white pigment, is a prototypical example of this category. However, the terminology “low toxicity” is currently a subject of intense scientific and regulatory debate. As detailed in the ECETOC Technical Report 122 and further elaborated by Bevan et al. and Riediker et al., critics argue that the term “low toxicity” is misleading for nanomaterials [28,29,30]. Riediker et al. emphasized that even chemically inert particles can exhibit significant biological reactivity due to their surface properties, supporting the shift to the term “Poorly Soluble Particles (PSPs)” to avoid implying safety [30]. Consequently, this alternative term, “Poorly Soluble Particles (PSPs)”, has been proposed to more accurately reflect their toxicological properties, which are driven primarily by particle accumulation rather than intrinsic chemical reactivity. Furthermore, the classification of TiO_2_ as a possible human carcinogen (Group 2B) by the IARC has challenged the traditional perception of its inertness.

Historically, definitive cases of human pneumoconiosis caused solely by TiO_2_ have been rare. However, a recent crucial case report from Japan has provided clearer insight into the human pathological response. Koyanagi and Kishimoto (2023) reported a case of pneumoconiosis in a worker exposed to high-purity (>95%) TiO_2_ powder (mean diameter 210 nm) for approximately 23 years [31]. Detailed pathological examination revealed centrilobular dust deposition mainly around pulmonary arteries and bronchioles, accompanied by mild fibrosis. Importantly, this presentation corresponds to the formation of Dust Macules (DM) progressing to Mixed Dust Fibrosis (MDF), rather than the classic silicotic nodules or neoplastic changes. This evidence suggests that the primary phenotype of TiO_2_-induced lung disease in humans—at least under occupational settings—lies along the DM–MDF axis, manifesting as an interstitial lung disease characterized by particle retention and mild stromal reaction.

Highly cytotoxic particles, such as crystalline silica, induce rapid cell death and complex inflammatory cascades that can obscure the fundamental physiological patterns of particle deposition and retention. In contrast, because TiO_2_ NPs possess low intrinsic toxicity, they serve as an ideal probe to investigate the biological variables of deposition sites and clearance mechanisms. By using such biologically quiet particles, we can more accurately dissect the anatomical and physiological species differences between humans and rodents without the confounding factors of massive tissue destruction.

Therefore, the central question of this review is not merely about carcinogenicity, but about comparative pathology: Does the accumulation of TiO_2_ in rodent lungs replicate the human DM–MDF axis? Or do the anatomical differences create distinct responses? While toxicology has successfully established dose–response relationships and clearance kinetics, the precise architectural location of where particles reside has traditionally received less attention. In this review, we initiated a “Pneumoconiosis Renaissance”. We demonstrate that rats develop a species-specific “Airspace-Dominant Phenotype” that physically superimposes upon and masks the human-relevant interstitial events. By integrating recent data—including critical recovery studies that unmask the true pathology—we propose a new framework. We argue that future risk assessments must incorporate “Spatial Resolution”, rigorously distinguishing between the “Airspace Mask” (rat-specific overload) and the “Interstitial Truth” (human-relevant pneumoconiosis) to accurately bridge the interspecies gap.

### 1.6. Prospectus

To systematically decode this complex landscape, this review is structured as follows. Section 2 provides a critical re-evaluation of historical and recent animal inhalation studies, defining the species-specific “Spatial Phenotypes” and establishing the concept of the “Airspace Mask” in rats. Section 3 integrates these pathological insights into the Adverse Outcome Pathway (AOP) framework, proposing a necessary split of the inflammatory Key Event (KE3) into alveolar (rat-dominant) and interstitial (human-relevant) components. Section 4 discusses the practical implications for regulatory risk assessment, introducing a “Dual-Track” strategy and examining the generalizability of this framework to other PSLTs such as carbon black. Finally, Section 5 concludes with future perspectives, addressing the essential role of “Ground Truth” pathology in the era of AI and digital toxicology.

This article is not intended as a comprehensive or systematic listing of all studies related to TiO_2_ or poorly soluble (low-toxicity) particles.

Rather, this critical review focuses on seminal and industry-defining studies that have historically shaped the interpretation of particle-induced lung pathology in humans and experimental animals.

These core studies were selected based on their enduring influence on regulatory risk assessment, frequent citation in authoritative monographs and guidelines, and their central role in establishing prevailing pathological paradigms.

By re-examining these foundational studies in conjunction with our recent experimental and pathological findings, this review aims to identify a previously overlooked directional vector in particle-induced lung disease.

In doing so, we reassess long-standing assumptions regarding spatial pathology, species differences, and the interpretation of adverse outcome pathways associated with particle exposure.

## 2. Experimental Studies in Animal Models for TiO_2_: Decoding the Pathological Signal

To provide a comprehensive overview of the toxicological landscape, we summarized the key inhalation studies on TiO_2_ in mice (Table 2) and rats (Table 3). As detailed in these tables, we re-evaluated the reported pathological findings through the lens of our proposed “Spatial Phenotype”. A critical review of these historical data revealed a consistent pattern: while mouse studies consistently demonstrate a lack of specific architectural remodeling (Table 2), rat studies frequently report severe fibrosis and inflammation (Table 3). However, a common challenge in interpreting historical reports is that these lesions were often broadly categorized under generic terms like ‘fibrosis’, without distinguishing whether they were airspace-dominant (pulmonary dust foci: PDF) or interstitial (DM/MDF), often lumping them under generic terms like fibrosis. Notably, as highlighted in Table 3, studies incorporating extended recovery periods (e.g., Schaudien et al. [32]) provide pivotal evidence for “Interstitial Unmasking”, successfully differentiating transient airspace stagnation from persistent interstitial sequestration. This lack of spatial resolution in historical data has been a major source of confusion in risk assessment.

Since the late 20th century, numerous inhalation studies using titanium dioxide (TiO_2_) particles have been conducted. However, historically, these studies have been heavily dominated by a carcinogenicity-centric perspective, often overshadowing the evaluation of TiO_2_-induced lung disease as a form of pneumoconiosis.

The landmark study by Lee et al. (1985) first demonstrated that chronic inhalation of fine TiO_2_ particles induced bronchoalveolar adenomas and squamous cell carcinomas in rats [37]. Subsequently, Heinrich et al. (1995) revealed that TiO_2_ NPs possessed significantly higher carcinogenic potency per unit mass than fine particles [34]. Based largely on these rat inhalation studies, the International Agency for Research on Cancer (IARC) classified TiO_2_ as a Group 2B carcinogen (possibly carcinogenic to humans) [41].

Morrow (1988) proposed the concept of lung overload, a condition where impaired macrophage-mediated clearance leads to persistent inflammation and secondary genotoxicity [42]. Oberdörster et al. (1994) further refined this by proposing the surface area hypothesis, positing that particle surface area is the key determinant of overload [43]. Regarding species differences, Warheit, Bermudez, and colleagues (2002, 2004) conducted comprehensive comparative studies exposing rats, mice, and hamsters to equivalent concentrations of TiO_2_ (both fine TiO_2_ and TiO_2_ NPs) in subchronic 13-week inhalation studies followed by recovery periods of up to one year [35,36]. Their findings were definitive regarding the disparity in response: while rats exhibited severe, persistent inflammation and septal fibrosis—pathological precursors to tumorigenesis—under overload conditions, mice and hamsters showed only mild, transient responses and effective clearance, with no significant progressive fibrosis. This extreme sensitivity of rats is widely attributed to the lung overload phenomenon, characterized by the impairment of macrophage-mediated clearance [42,43].

These classical studies successfully established the hierarchy of sensitivity (Rat >> Mouse > Hamster), a concept comprehensively analyzed by Warheit et al. (2016) [44]. In their review, they detailed how rats exhibited a high degree of alveolar epithelial proliferation and fibrosis under overload conditions, whereas mice and hamsters—even at equivalent burdens—showed minimal to low responses. However, these studies left a critical gap in pathological understanding. The discussions focused almost exclusively on tumor incidence and clearance kinetics, treating the underlying tissue changes merely as non-specific fibrosis or chronic inflammation. Crucially, these toxicological evaluations failed to assess the lesions through the lens of pneumoconiosis pathology established in humans (as described in Section 1). Did the overloaded macrophages form Dust Macules (DM)? Did the fibrosis organize into Nodules? Or did it progress to Mixed Dust Fibrosis (MDF)? Because previous studies lacked this architectural granularity—distinguishing between alveolar airspace, alveolar interstitium, and bronchiolar interstitium—the direct link between rat overload and human pneumoconiosis has remained obscure.

Therefore, to accurately evaluate the human relevance of TiO_2_, we must shift our focus from carcinogenicity back to pneumoconiosis. In the following subsections, we review recent studies that have revisited these animal models with rigorous histopathological analysis, mapping the precise distribution of particles and cells to determine whether they replicate the human DM–MDF axis.

Furthermore, in the current toxicological landscape emphasizing the 3Rs (Replacement, Reduction, Refinement) and New Approach Methodologies (NAMs), the necessity of in vivo studies is often scrutinized. However, current in vitro and in silico models still struggle to fully replicate the complex spatiotemporal dynamics of the living lung, such as mucociliary clearance, lymphatic drainage, and long-term architectural remodeling. Consequently, providing a rigorous, high-resolution histopathological dataset from animal models serves a dual purpose: it not only elucidates the true biological nature of PSLT-induced pneumoconiosis but also provides the indispensable “Ground Truth” required to validate and refine future alternative testing strategies.

### 2.1. Detailed Histopathological Analysis in Mouse Models: Absence of the Dust Macule Architecture

Historically, comparative studies have consistently indicated that mice are remarkably resistant to particle-induced lung toxicity compared to rats. As noted above, Bermudez et al. demonstrated effective clearance and minimal tissue response in mice [35,36]. Reinforcing this, Warheit et al. (2016) [44] highlighted that while mice do develop pulmonary inflammation under lung overload conditions, they lack the progressive fibroproliferative sequelae seen in rats. This inherent biological resistance explains why mice fail to develop complex architectural lesions. However, these seminal reports did not specifically address the architectural organization of retained particles.

To rigorously evaluate the pathological phenotype in mice, Yamano et al. (2022) conducted a comprehensive 26-week whole-body inhalation study using rasH2 mice [33]. Despite exposure to a high concentration (32 mg/m^3^) of anatase TiO_2_ NPs sufficient to induce lung overload in rats, the results were striking in that there was no evidence of damage to the lung in mice. Detailed histopathological mapping revealed that while particle-laden macrophages were present, they remained scattered or formed only loose aggregates within the alveolar spaces. Crucially, these aggregates did not organize into the distinct topography-specific structures defined as Dust Macules (DM) in human pathology. There was no evidence of the peribronchiolar interstitial accumulation or the architectural remodeling required for diagnosis of a macule. This suggests that mice possess a biological mechanism that prevents the architectural reorganization of particle-laden macrophages into pneumoconiotic foci. Consequently, while mice serve as a useful negative control for carcinogenicity, they fail to replicate the DM–MDF axis observed in human pneumoconiosis.

### 2.2. The Rat-Specific Overload Phenotype: The Challenge of Biological Masking

While rats are the most sensitive species, the lack of spatial differentiation in historical studies has made it difficult to fully contextualize the human relevance of these lesions. The comparative inhalation studies by Bermudez et al. (2002, 2004) [35,36] serve as the global benchmark, utilizing a robust design with a 13-week exposure period and recovery periods of up to 52 weeks. Historically, the severe lesions observed in high-dose rats were described as progressive fibroproliferative lesions or alveolar epithelial metaplasia.

However, applying our Pneumoconiosis Renaissance criteria to their published histological images revealed a new perspective. We retrospectively identified that the lesion reported by Bermudez et al. (2002) corresponded morphologically to Fibrotic Pulmonary Dust Foci (fPDF), characterized by cholesterol granulomas and fibrosis of the alveolar septa (Narrow Interstitium). This distinction is vital: while Bermudez et al. correctly identified fibrosis, it was primarily located in the alveolar walls—a rat-specific overload response—rather than the peribronchiolar Broad Interstitium characteristic of human pneumoconiosis induced by dusts like coal or TiO_2_. Similarly, the histological findings reported by Bermudez et al. (2004) show a typical early-stage Pulmonary Dust Foci (PDF), consisting of alveolar macrophage aggregates.

To define this pathology, Yamano et al. proposed the “PDF to fPDF axis” [38,39]. Unlike human interstitial macules, these lesions are characterized by the massive filling of alveolar airspaces with inflammatory cells and collagen—a phenomenon we term “Airspace Stagnation”. Morphologically, this rat-specific response closely resembles the “Alveolar Filling Disorders” (e.g., Alveolar Macrophage Pneumonia) defined in the latest human clinical classification [45], rather than true interstitial pneumoconiosis. Crucially, the recent 2-year study by Yamano and Umeda [39] revealed a critical dichotomy. While severe fibrosis was exclusively driven by the airspace-originating fPDF, rats were also found to form Dust Macules (DM) in the subpleural and perivenous interstitium. Importantly, unlike human DMs, which often progress to collagenous nodules, these rat DMs remained non-fibrotic and architecturally quiescent (or non-progressive), even after two years.

Critically, this lack of progression should be viewed as a validation of the model’s specificity rather than a limitation. Since pure TiO_2_ is a PSLT with low inherent fibrogenicity, a progression to massive fibrosis (MDF) in rats would represent a species-specific exaggeration of fibrogenicity relative to human epidemiological reality (where DM predominates). The rat model correctly distinguished the “tattoo” (DM) from the “scar” (MDF). We postulate that if this “Spatially Resolved” model was challenged with high-toxicity mixed dusts (e.g., silica-containing), these “Unmasked” DMs would likely progress to MDF, demonstrating the model’s capacity to discriminate true hazard potency.

This leads to a pivotal hypothesis: The rat-specific Airspace-Dominant Phenotype (PDF/fPDF) acts as a Biological Mask. Although rats possess the anatomical potential to form human-relevant interstitial DMs, this response is quantitatively overshadowed by the overwhelming toxicity of the airspace-dominant fPDF. The unceasing particle influx in continuous exposure models perpetuates intense airspace inflammation, creating a pathological overlay that obscures the subtle, human-relevant interstitial remodeling.

#### The Molecular Basis of the “Airspace Mask”: Insights from Comparative Transcriptomics

Beyond anatomical differences, intrinsic species-specific macrophage biology serves as the molecular engine driving the “Airspace Mask”. A recent pivotal study by Perez et al. [46] provided the missing link by comparing the transcriptomic responses of primary rat and human alveolar macrophages (isolated via bronchoalveolar lavage) to TiO_2_ and carbon black (Printex 90) overload in vitro. By utilizing primary cells rather than cell lines, this study provides a high-fidelity representation of the “Airspace” compartment.

Their findings revealed a striking contrast: while primary rat macrophages exhibited massive transcriptional dysregulation involving thousands of genes in response to both PSLTs, primary human macrophages remained remarkably quiescent, showing minimal to no significant gene modulation [46]. Crucially, the “Rat-Specific Overload Signature” identified in these primary cells functionally explains the formation of Pulmonary Dust Foci (PDF). Specifically, rat macrophages upregulated Cxcr4, a key driver of neutrophil recruitment, which correlates with the severe alveolitis (KE3-a) observed in rats. Simultaneously, they downregulated genes essential for efferocytosis, such as Cfp, C1qb, and C1qc. This suppression of clearance pathways likely leads to the accumulation of cellular debris within the alveoli, providing the molecular basis for “Airspace Stagnation”.

Thus, while absolute macrophage numbers remain difficult to compare reliably across species due to methodological variability, differences in macrophage functional programming appear to be a more critical determinant of overload pathology. In contrast, the “Transcriptomic Quiescence” of primary human macrophages suggests that human alveolar macrophages fail to mount this molecular response, allowing particles to gradually translocate into the interstitium without triggering the intense alveolar inflammatory cascade seen in rats. Thus, the “Biological Mask” is not a passive artifact but an active, rat-specific pathological cascade driven by hyper-reactive macrophages.

### 2.3. Unmasking the Truth: The Role of Recovery and Spatial Resolution

If the “Airspace Mask” obscures the truth, how can we see what lies beneath? The key lies in experimental designs that allow the Mask to fade or allow sufficient time for interstitial migration.

Our comparative analysis of the 13-week [38] and 2-year [39] studies by Yamano et al. highlights a critical temporal factor: Dust Macules (DMs) were absent at 13 weeks but clearly present after 2 years. This suggests that the formation of human-relevant interstitial lesions is a slow process. In standard subchronic bioassays, the “Interstitial Truth” has not yet matured, or is completely obscured by the rapid-onset “Airspace Mask” (PDF).

To overcome this temporal barrier, the recent comparative study by Schaudien et al. (2025) provides critical insights [32]. Using a nose-only inhalation system, they exposed rats to TiO_2_ NPs for 28 days followed by a substantial 94-day recovery period. Consistent with the high sensitivity of rats, initial lesions appeared as alveolar PDF-like lesions (the Mask). However, unlike continuous exposure models, the extended recovery period allowed the acute alveolar inflammation to subside.

This “Unmasking Strategy” proved successful. When re-evaluated through the lens of the Broad Interstitium anatomy (Section 1.3), particle-laden macrophages in the Schaudien study were clearly identified aggregating in the perivenous interstitium (functionally equivalent to human interlobular septa). This finding is of paramount importance. It demonstrates that rats do possess the biological potential to form the DM–MDF axis, but this interstitial phenotype is often missed in standard studies because it is visually overwhelmed by the concurrent airspace pathology. Human pathology reviews, such as Warheit et al. (2016) [44], vividly illustrate the characteristic interstitial sequestration of particulate material within the broncho-vascular bundles of coal miners, which serves as a benchmark for human-relevant dust macules. Rodents lack the interlobular septa seen in humans (Table 1; Figure 2A,B), meaning that the perivenous interstitium [20] is the only homologous site in rats and humans for lymphatic drainage and macule formation. Consequently, sequestration of particulate material within the perivenous interstitium in the rat lung allows the formation of “human-relevant” dust macules in rats.

The specific localization of these rodent lesions—predominantly perivenous rather than axial—reflects the fundamental species difference in lymphatic architecture. While human DMs typically favor the axial (peribronchiolar) interstitium, visualization studies using Prox1-GFP reporter rats [47] and mice [48] have revealed that rodent pulmonary lymphatics are abundantly distributed along the pulmonary veins but are relatively sparse or discontinuous in the peribronchiolar regions. Consequently, the distinct perivenous sequestration observed in the ‘Unmasked’ rat model represents the physiological drainage into their primary lymphatic network, functionally mirroring the heavy axial drainage seen in humans.

Therefore, the findings from Yamano et al. (continuous exposure) [39] and Schaudien et al. (recovery after exposure) [32] are not contradictory but complementary regarding the kinetics of interstitial translocation. Yamano et al. demonstrated that under continuous overload, interstitial sequestration (DM formation) does occur but is a slow process heavily obscured by the dominant airspace pathology (the Mask). In contrast, Schaudien et al. showed that introducing a recovery period allows this “Airspace Mask” to subside, thereby accelerating the visibility of the interstitial marker. Thus, Yamano et al. confirmed the biological potential for DM formation in rats, while Schaudien et al. provided the optimal methodology to visualize DM formation in rats for risk assessment.

We propose the “Alveolar Traffic Jam” hypothesis to explain this dynamic. Under continuous exposure, the unceasing influx of particles creates an overwhelming alveolar load, potentially congesting the physiological drainage routes (the Broad Interstitium) and physically blockading the lymphatic access points. Consequently, the standard continuous protocol might ironically act as a barrier to interstitial translocation. The “recovery period”, therefore, is not merely a passive wait time but an active physiological restoration. Stopping the exposure clears the alveolar congestion, allowing the “Slow Translocator” phenotype of rats to finally sequester particles into the interstitium effectively. Crucially, recent findings by Lee et al. provide empirical support for this view; they demonstrated that the experimental depletion of alveolar macrophages in rats significantly accelerated the translocation of carbon black nanoparticles to the lung-associated lymph nodes [49]. This suggests that the alveolar macrophage burden itself acts as a functional blockade to interstitial drainage.

## 3. Integrating Pathology into the AOP Framework: The Missing Spatial Dimension

In the modern era of toxicology, the transition to mechanism-based risk assessment relies heavily on the Adverse Outcome Pathway (AOP) framework [50]. A fundamental design philosophy of the AOP is “modularity”—the creation of generic Key Events (KEs) that can be reused across different organs and stressor types [51]. While this abstraction facilitates the development of high-throughput in vitro assays, it comes with an unintended consequence: it strips away the “spatial context”.

For soluble chemicals, where molecular signaling often dictates toxicity, this abstraction may be acceptable. However, for insoluble particles like TiO_2_, where the particle resides (anatomy) essentially dictates how the tissue responds (mechanism).

For TiO_2_ NPs, Braakhuis et al. (2021) established the current benchmark AOP [52], outlining the cascade from impaired clearance (Initiating Event) to lung tumors (Adverse Outcome). They defined critical Key Events (KEs), including ROS generation (KE1), oxidative stress (KE2), persistent inflammation (KE3), and epithelial cell injury/proliferation (KE4/KE6). We fully agree that this cascade is operative, particularly regarding the molecular drivers. Similarly, Warheit et al. (2016) [44] provided a comprehensive review that also included a discussion of an AOP concept to explain rat-specific lung tumor development. However, a critical limitation remains: both frameworks largely lack the mandatory dimension of “Spatial Resolution”.

In the current framework, Inflammation (KE3) is treated as a single functional block. This generalized approach, while useful for high-throughput screening, may not fully align with the evolving stratification in clinical medicine. As emphasized in the newly updated international multidisciplinary classification of interstitial pneumonias (ERS/ATS 2025) [45], the fundamental stratification of lung injury now explicitly distinguishes between “Interstitial disorders” and “Alveolar filling disorders”. Crucially, the Airspace-Dominant inflammation seen in rat lung overload (PDF–fPDF axis) morphologically mirrors the newly defined category of “Alveolar Macrophage Pneumonia (AMP)” within the alveolar filling disorders. In stark contrast, the Interstitial pathology driving human pneumoconiosis (DM–MDF axis) aligns with the Bronchiolocentric Interstitial Pneumonia (BIP) pattern, characterized by airway-centered inflammation and fibrosis within the interstitial disorders category. By grouping these biologically distinct pathologies—Rat AMP-like responses and Human BIP-like responses—under generic labels, the current AOP inadvertently allows the rat-specific “Pathological Noise” to be conflated with human-relevant hazards.

**Figure 5:**
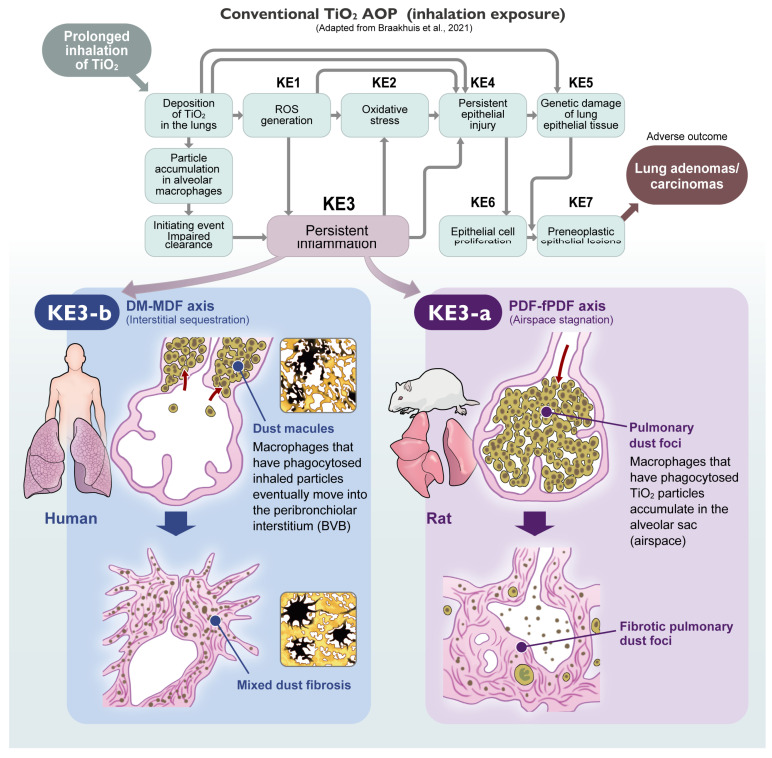
Divergent Pathogenesis and the “Split-AOP” Concept. (**Left**) Human Scenario (The Signal): Driven by “Interstitial Sequestration” (DM–MDF axis). Inhaled biodurable particles translocate to the Broad Interstitium (specifically the peribronchiolar interstitium of the BVB). Macrophages accumulate within the interstitial connective tissue (Dust Macules; DM), triggering stromal remodeling and stellate fibrosis (Mixed Dust Fibrosis; MDF). This corresponds to KE3-b: Chronic Interstitial Inflammation. Inset images show the representative macroscopic/histological appearance of DM and MDF (Source: JOHAS Comprehensive Atlas). (**Right**) Rat Scenario (The Noise): Driven by “Airspace Stagnation” (PDF–fPDF axis). Under overload conditions, particle-laden macrophages accumulate within the alveolar airspaces (Pulmonary Dust Foci; PDF). Fibrosis develops primarily within the airspace (alveolar septal fibrosis), physically obstructing the lumen (Fibrotic PDF; fPDF). This corresponds to KE3-a: Chronic Alveolar Inflammation. Note: Standard toxicological assessments often conflate these two distinct biological responses under the generic term “fibrosis”. This schema illustrates the two distinct morphogenic pathways of PSLT-induced lesions and their integration into the refined Adverse Outcome Pathway (AOP). The upper panel displays the conventional TiO_2_ AOP framework adapted from Braakhuis et al. [52].

To achieve the Pneumoconiosis Renaissance, future AOPs must undergo a structural evolution. We propose that KE3 (Persistent Inflammation) must be spatially segregated into two distinct sub-events (Figure 5):

1. **KE3-a: Chronic Alveolar Inflammation (Rat-Dominant):**

Corresponds to “Airspace Stagnation” and PDF formation. This is the primary driver of the rat-specific overload phenotype. It serves as a highly sensitive, pragmatic screening marker (including BALF analysis) for establishing conservative exposure limits (OELs). However, since its mechanistic relevance to humans is limited, it should not be pursued as a model for human interstitial fibrosis.

2. **KE3-b: Chronic Interstitial Inflammation (Human-Relevant):**

Corresponds to the sequestration of particles in the “Broad Interstitium” (DM–MDF axis). This is the critical driver for human pneumoconiosis. As shown by Schaudien et al. [32], identifying this KE in rats requires Unmasking strategies (e.g., recovery periods) to filter out the noise of KE3-a.

By incorporating this distinction, the relationship between KEs becomes clearer.

- In Rats: The pathway is often dominated by IE (Impaired Clearance) → KE3-a (Alveolar Inflammation) → KE6 (Epithelial Proliferation) → AO (Tumors).
- In Humans: The pathway of concern is likely IE (Translocation) → KE3-b (Interstitial Inflammation) → AO (Interstitial Fibrosis/Pneumoconiosis).

Without this distinction, risk assessments rely on a “mixed signal”. By adopting this spatially resolved AOP, toxicologists can utilize the rat model more effectively: utilizing KE3-a for hazard identification (Overload alert) while specifically seeking KE3-b for human risk characterization.

## 4. Implications for Risk Assessment and Future Directions

### 4.1. Integrating Spatial Resolution into Risk Assessment: A Dual-Track Approach

Riediker et al. (2019) comprehensively reviewed the state of particle toxicology, rightfully emphasizing the importance of appropriate dosimetry (e.g., surface area over mass) [30]. However, while the field has clarified “how much” and “in what form” particles cause toxicity, the dimension of “where”—the spatial distribution—has remained less explored. Our proposed refinement of the AOP (splitting KE3 into KE3-a and KE3-b) has profound implications for occupational health and safety.

Currently, regulatory bodies establish Occupational Exposure Limits (OELs) largely based on the most sensitive endpoint in animals. In the case of TiO_2_, this is often the rat-specific “Airspace Stagnation” (KE3-a: Alveolar Inflammation) or its sequelae (fPDF). From a worker protection standpoint, adopting the most sensitive endpoint—even if it represents a “Rat-Specific Overload Phenotype”—is a prudent and pragmatic approach to prevent lung overload. We do not advocate for relaxing these limits if they successfully prevent dust accumulation in the lung.

However, for Hazard Characterization and Classification (e.g., carcinogenicity labeling), relying solely on KE3-a may result in an overly conservative or confounded hazard characterization. It treats the “Airspace Mask” as the toxicological target equivalent to human disease. To resolve this, we propose a “Toxicological Triage” approach implemented via a “Dual-Track” Assessment Strategy:

1. **Track 1 (Screening & Prevention):** Use KE3-a (Chronic Alveolar Inflammation) as a sensitive and pragmatic warning signal (e.g., BALF analysis). If a substance induces KE3-a in rats, it indicates a potential for biopersistence and lung overload. Regulatory limits should be set to prevent this state. However, deep mechanistic investigation of this phenotype is not recommended for human extrapolation as it carries a high risk of being a species-specific anomaly.
2. **Track 2 (Human Extrapolation):** Use KE3-b (Chronic Interstitial Inflammation) for definitive hazard classification and mechanistic elucidation. To determine if a substance poses a risk of human pneumoconiosis or interstitial fibrosis, data must be evaluated for interstitial changes. This represents the true pathological ground for investigating human disease mechanisms. As demonstrated by Schaudien et al., this requires “Unmasking” strategies—specifically, exposure designs with extended recovery periods that allow the airspace noise to subside.

It is noteworthy that existing regulatory guidelines, such as OECD TG 413 (Option B for poorly soluble solid aerosols) [53], already recommend the inclusion of satellite recovery groups to evaluate lung burden and clearance kinetics. We argue that these existing protocols hold untapped potential. While the guideline focuses on quantitative clearance, our Pneumoconiosis Renaissance reinterprets this recovery phase as a qualitative filtering window. By utilizing the post-exposure observation period mandated by Option B in OECD TG 413, pathologists can detect the Unmasked Interstitial Signal (KE3-b) without the need for novel, non-standardized experimental designs.

### 4.2. Broadening the Scope: From TiO_2_ to All PSLT

While this review primarily focused on TiO_2_ as the prototypical PSLT, the identified pathological mechanism—the Airspace Masking driving of the PDF–fPDF axis—is likely not unique to TiO_2_, but rather a Class Effect applicable to all PSLTs. Since this phenotype is fundamentally driven by the physical state of lung overload and the distinct lymphatic clearance pathways dictated by species-specific lung architecture (i.e., the functional implications of the presence or absence of secondary lobules) rather than specific chemical toxicity, we postulate that the “Spatial Phenotype” represents a universal biological response to inhaled biodurable particles. It is highly probable that other major granular biodurable particles, such as carbon black and toner, follow a similar morphogenic pathway in rats.

Recent transcriptomic evidence supports this generalization. As discussed in Section 2.2, Perez et al. (2025) demonstrated that rat alveolar macrophages exhibit an almost identical pro-inflammatory profile (e.g., Cxcr4 upregulation, C1q downregulation) when exposed to either TiO_2_ or carbon black (Printex 90) [46]. This confirms that the rat-specific “Airspace Stagnation” is a universal response to the pulmonary overload of biodurable particles. These parallels indicate that the Spatial Phenotype framework is not TiO_2_-specific but represents a class effect of biodurable PSLTs under lung overload conditions.

It is important to distinguish these biodurable PSLTs from partially soluble nanomaterials, such as amorphous silica. While amorphous silica is industrially relevant, its higher solubility in biological fluids can lead to faster clearance rates compared to TiO_2_ or carbon black, potentially altering the kinetics of “Mask” formation and interstitial translocation. Therefore, while our “Spatial Phenotype” framework provides a robust model for biodurable dusts, the applicability to materials with intermediate solubility requires further specific investigation to decouple chemical toxicity from physical retention effects.

Historically, toxicological evaluations of these substances have broadly been described lesions as “alveolar accumulation” or “septal fibrosis” without the necessary spatial resolution. This lack of distinction may have similarly conflated rat-specific noise with human risk. Therefore, we propose that the Pneumoconiosis Renaissance can serve as a valuable framework for evaluating this entire class of materials. We anticipate that a retrospective histopathological re-evaluation of archived tissue samples for carbon black, toner, and other PSLTs—applying the specific criteria of KE3-a vs. KE3-b—would be a promising avenue for future research, offering a renewed perspective on existing safety data.

### 4.3. The “Ground Truth” for Future Methodologies

Finally, we must address the role of in vivo studies in the era of the 3Rs (Replacement, Reduction, Refinement) and New Approach Methodologies (NAMs). There is increasing pressure to replace animal inhalation studies with in vitro lung models. However, current technologies (e.g., lung-on-a-chip) primarily model the alveolar–capillary barrier (Narrow Interstitium) and often lack the complex “Broad Interstitium” (axial/peribronchiolar tissues) and lymphatic drainage systems essential for pneumoconiosis development. We cannot model what we do not fully understand. Recent in vivo kinetic studies continue to highlight the complexity of TiO_2_ retention and lymphatic translocation [54], while McCormack et al. emphasized how dispersion protocols alter these physicochemical identities [55]. Without accounting for these “input” and “transit” variables defined in vivo, in vitro models remain incomplete.

Therefore, the goal of the Pneumoconiosis Renaissance is not merely to revisit historical data, but to provide the “Ground Truth” necessary to build sophisticated in vitro models. Just as Gough–Wentworth sections historically resolved the confusion around coal dust toxicity by visualizing the spatial reality [10], elucidating the precise histogenesis of lesions today—specifically distinguishing between the Airspace Mask (KE3-a) and the Interstitial Truth (KE3-b)—provides the architectural blueprint that future NAMs must replicate to be biologically valid. Only by thoroughly confronting the pathology in in vivo models can we establish a truly scientific basis for alternative testing.

## 5. Conclusions and Perspectives

A conceptual framework for the future of nanotoxicology.

- (The Filter) Spatiotemporal Resolution: By applying anatomical and chronological perspectives (e.g., recovery periods), we can separate the data into two tracks.
- Track 1 (Screening & Prevention/KE3-a): Represents Airspace Stagnation (PDF/fPDF). Use as a sensitive warning signal for establishing OELs (pragmatic approach).
- Track 2 (Human Extrapolation/KE3-b): Represents Interstitial Sequestration (Dust Macule). This is the basis for definitive hazard classification and mechanistic elucidation.

Historically, the safety assessment of TiO_2_ and other PSLTs has focused primarily on carcinogenicity and lung overload, often leaving the detailed pathological identity of the lesions in a “Spatial Blind Spot”. Through rigorous histopathological mapping of rodent models, we have demonstrated a critical dichotomy: while mice remain largely unresponsive, rats develop a unique “Airspace-Dominant Phenotype” (PDF–fPDF axis). This phenotype, driven by species-specific airspace stagnation, acts as a “Biological Mask” (KE3-a) that superimposes upon and obscures the human-relevant “Interstitial Phenotype” (DM–MDF axis/KE3-b).

**Figure 6:**
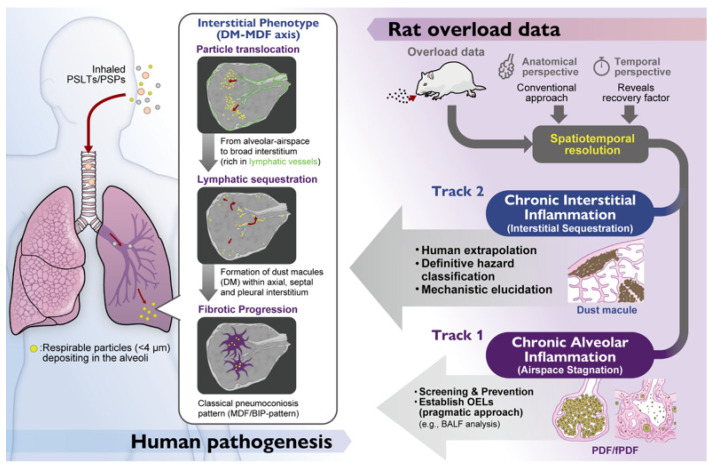
The Pneumoconiosis Renaissance: Filtering out “Pathological Noise” through Spatiotemporal Resolution. (**Left Panel**) Human Pathogenesis: Illustrates the “Interstitial Phenotype” (DM–MDF axis). Inhaled particles undergo translocation from the alveolar airspace to the broad interstitium (rich in lymphatic vessels), leading to lymphatic sequestration (Dust Macules) and progression to fibrotic BIP patterns (MDF). (**Right Panel**) The Dual-Track Strategy: Illustrates how to decode Rat Overload Data. (Input) The Chaos: Standard rat data often presents a conflated signal where species-specific airspace pathology masks human-relevant interstitial events.

We propose that the next generation of nanotoxicology must evolve from macroscopic outcome analysis to microscopic spatial profiling. We must look beyond the alveolar noise to hear the quiet, interstitial signal. To hear the true signal of human risk, we must learn to decode the rat model (Figure 6). This requires nothing less than a Pneumoconiosis Renaissance: a return to architectural understanding to guide the future of AOP development. By introducing spatial resolution, specifically distinguishing key alveolar-air space and narrow-interstitial events from broad-interstitial events and by employing study designs that enable unmasking (such as extended recovery periods), the rat model can be transformed from a source of confusion into a refined and informative tool for protecting worker health.

### The Role of AI and Digital Pathology: The Necessity of “Ground Truth”

The rapid evolution of Artificial Intelligence (AI) and Machine Learning (ML) offers a promising avenue for high-throughput toxicity screening. However, in the context of particle-induced lung pathology, technology cannot precede biology. As of 2026, a fundamental challenge remains: the lack of precise pathological annotation based on comparative anatomy and lymphatic clearance physiology. Current datasets often label rat-specific “Airspace Stagnation” (PDF) simply as “fibrosis”, conflating it with human-relevant “Interstitial Remodeling” (DM/MDF). Applying AI to such confounded data will only reinforce existing biases (“Garbage In, Garbage Out”). Therefore, the “Pneumoconiosis Renaissance” is not merely a revisit of old concepts but a necessary step to establish the “Ground Truth” for future technologies. Before we can leverage AI to separate the “Signal” from the “Noise”, we must first teach the algorithms—and the toxicologists—how to anatomically distinguish the Broad Interstitium from the Airspace. Only with this verified “Spatial Phenotype” can Digital Pathology truly bridge the interspecies gap.

Moreover, this spatially resolved framework provides more than an interpretive advance for current PSLTs. It offers a universal “Rosetta Stone” to decode the pathological signals of generic dusts—ranging from legacy materials like carbon black and toner to future advanced materials. By anchoring hazard identification in the core biology of pneumoconiosis, this approach equips regulatory agencies with a forward-compatible methodology capable of detecting health risks arising from future, unprecedented chemistries. In this sense, the Pneumoconiosis Renaissance is not merely a reinterpretation of past data but a scientific investment in preparedness, enabling public health and regulatory systems to anticipate and respond to the challenges posed by tomorrow’s advanced materials.

## Figures and Tables

**Table 1:** Comparative Pathology of Pneumoconiosis: Human vs. Rat (adapted from Ref. [8]).

Feature	Human (Gold Standard)	Rat (Overload Model)
Anatomy (Secondary Lobule)	Present (Well-developed interlobular septa)	Absent (No interlobular septa; continuous parenchyma)
Lymphatic Drainage Route	Axial & Interlobular (Broad Interstitium)	Axial & Perivenous (Functional equivalent)
Primary Response Site	Interstitial (Peribronchiolar/Interlobular)	Airspace (Alveolar duct/Alveoli)
Key Lesion (Initial)	Dust Macule (DM)—Interstitial sequestration	Pulmonary Dust Foci (PDF)—Airspace stagnation
Key Lesion (Advanced)	Mixed Dust Fibrosis (MDF)/PMF	Fibrotic PDF (fPDF)—Mimics PMF but airspace-derived
Impact on Risk Assessment	Standard for chronic pneumoconiosis	“Biological Mask” (Confounding factor)

This table highlights the fundamental anatomical and pathological discrepancies that necessitate the “Pneumoconiosis Renaissance”. Note that while humans possess well-developed interlobular septa facilitating interstitial sequestration (Dust Macule), rats lack these structures, leading to airspace stagnation (PDF), which acts as a “Biological Mask” in risk assessment.

**Table 2:** Summary of chronic and subchronic inhalation studies in mice: absence of the “Dust Macule” architecture.

Reference	Animal	Material	Exposure Duration/Frequency	Concentrations	Recovery Period	Reported Findings (Original Authors)	Spatial Phenotype (Our Interpretation)
Yamano et al., 2022 Ref. [33]	CByB6F1-Tg(HRAS)2Jic (rasH2)	Anatase (30 nm, purity 97.9%)	26 weeks (6 h/day, 5 days/week)	2, 8, or 32 mg/m^3^	None	No evidence of carcinogenicity or fibrosis. Particles were phagocytosed by alveolar macrophages but these macrophages remained as scattered cells or loose aggregates within the alveolar airspaces. No distinct formation of “foci” or interstitial accumulation of macrophages was observed.	No architectural remodeling (non-responder): Unlike rats, mice lack the biological response to organize particle-laden macrophages into specific structures (neither PDF nor DM), even at high concentrations.
Heinrich et al., 1995 Ref. [34]	NMRI, C57BL/6N	P-25 (80% anatase, 20% rutile)	13.5 months (18 h/day, 5 days/week)	7.2, 14.8, or 9.4 mg/m^3^	Up to 9.5 months	No description of non-neoplastic lesions (focus on tumorigenicity).	Not specified: Lack of description regarding interstitial changes or macule formation.
Bermudez et al., 2002 Ref. [35]	B3C3F1 (female)	Pigmentary TiO_2_	13 weeks (6 h/day, 5 days/week)	10, 50, or 250 mg/m^3^	Up to 52 weeks	Mild alveolar type II cell hypertrophy without fibrosis. Inflammation persisted but was moderate.	Minimal response: Despite high concentrations, no distinct PDF or Macule formation observed.
Bermudez et al., 2004 Ref. [36]	B3C3F1 (female)	TiO_2_ NPs	13 weeks (6 h/day, 5 days/week)	0.5, 2.0, or 10 mg/m^3^	Up to 52 weeks	Particle-laden macrophages accumulated in centriacinar regions. Inflammation remained elevated but epithelial hyperplasia was mild.	Minimal response: Lack of progressive fibrosis or structural remodeling compared to rats.

Note that despite exposure to high concentrations sufficient to induce overload in rats, mice consistently failed to develop the structural organization of particle-laden macrophages into “Dust Macules” or progressive fibrosis.

**Table 3:** Summary of chronic and subchronic inhalation studies in rats: re-evaluating the “Airspace-Dominant Phenotype” and the unmasking of interstitial pathology.

Reference	Animal	Material	Exposure Duration/Frequency	Concentrations	Recovery Period	Reported Findings (Original Authors)	Spatial Phenotype (Our Interpretation)
Lee et al., 1985 Ref. [37]	SD (male/female)	Respirable TiO_2_ (1.5–1.7 μm)	24 months (6 h/day, 5 days/week)	10, 50, or 250 mg/m^3^	None	Minute collagenized fibrosis occurred in the alveolar walls enclosing large dust cell aggregates. Collagen deposition remained minimal.	Airspace-dominant (PDF): The ‘dust cell aggregates’ correspond to alveolar stagnation. No discussion of pneumoconiosis relevance.
Heinrich et al., 1995 Ref. [34]	Wistar (female)	TiO_2_ NPs (P25)	24 months (18 h/day, 5 days/week)	7.2, 14.8, or 9.4 mg/m^3^	Up to 6 months	Interstitial fibrosis progressed from very slight to moderate with 2 years of exposure.	Unverifiable: Lacks histological images to distinguish between septal thickening (airspace) and true interstitial fibrosis.
Bermudez et al., 2002 Ref. [35]	F344 (female)	Pigmentary TiO_2_	13 weeks (6 h/day, 5 days/week)	10, 50, or 250 mg/m^3^	Up to 52 weeks	“Progressive fibroproliferative lesions”, “alveolar epithelial metaplasia”, and “septal fibrosis” in high-dose rats.	Airspace-dominant (fPDF): Retrospective review of the histopathological findings reported by Bermudez et al. [35] confirms the characteristics of Fibrotic PDF (fPDF), including cholesterol granulomas and airspace fibrosis. Potential interstitial DMs were likely present but overshadowed by this overwhelming airspace pathology (Biological Mask).
Bermudez et al., 2004 Ref. [36]	F344 (female)	TiO_2_ NPs	13 weeks (6 h/day, 5 days/week)	0.5, 2.0, or 10 mg/m^3^	Up to 52 weeks	“Septal thickening” and “slight proliferation of Type II cells”. Minimal fibrotic response noted.	Airspace-dominant (PDF): Retrospective review of the histopathological findings reported by Bermudez et al. [36] identifies loose aggregates of particle-laden macrophages in the alveolar duct/alveoli, consistent with the definition of PDF (early stage). No distinct formation of interstitial DMs.
Yamano et al., 2022 Ref. [38]	F344 (male/female)	Anatase (30 nm, purity 97.9%)	13 weeks (6 h/day, 5 days/week)	6.3, 12.5, 25, or 50 mg/m^3^	None	Pulmonary dust foci (PDF): Focal aggregations of particle-laden macrophages localized in the proximal alveolar regions.	Airspace-dominant (PDF): Clearly defined as an airspace stagnation phenotype. Crucially, unlike the 2-year study, no interstitial Dust Macules (DM) were formed at this subchronic stage, indicating DM formation requires longer duration.
Yamano et al., 2025 Ref. [39]	F344 (male/female)	Anatase (30 nm, purity 97.9%)	24 months (6 h/day, 5 days/week)	0.5, 2, 8 mg/m^3^	None	Two distinct lesions developed: (1) Fibrotic PDF (fPDF): Advanced alveolar lesions with fibrosis, inflammation, and epithelial hyperplasia. (2) Dust Macules (DM): Interstitial accumulation of macrophages in subpleural/perivascular regions without fibrosis or inflammation. DMs appeared later than fPDFs.	Dual phenotype (airspace-dominant toxicity): Rats can form human-like interstitial DMs (confirming anatomical potential). However, toxic fibrosis (fPDF) is driven exclusively by “Airspace Stagnation”, which quantitatively overwhelms the benign interstitial DMs.
Schaudien et al., 2025 Ref. [32]	Wistar	NM-103/104/105 (Surface modified)	4 weeks (Nose-only)	3, 12, and 48 mg/m^3^	94 days	94-day recovery reduced alveolar inflammation. Particle-laden macrophages became prominent in the perivenous interstitium and BALT. No granuloma formation.	Interstitial unmasking: The removal of the “Airspace Mask” (acute inflammation) via recovery allowed the visualization of interstitial sequestration (macule formation) in the perivenous regions, equivalent to human interlobular septa.
Okada et al., 2019 Ref. [40]	Wistar (male)	TiO_2_ NPs (P25)	4 weeks	4.1 mg/m^3^	None	Mild pulmonary inflammation and thickened pulmonary alveolar wall.	Minimal response: Histological changes were subtle; difficult to categorize spatially based on provided images.

Historical studies often lumped alveolar and interstitial events. The “Spatial Phenotype” column re-interprets these findings based on the “Airspace Mask” theory. Note that studies with extended recovery periods (e.g., Schaudien et al.) successfully unmasked the interstitial phenotype relevant to human pneumoconiosis.

## Data Availability

No new data were created or analyzed in this study. Data sharing is not applicable to this article.

## Abbreviations

The following abbreviations are used in this manuscript:

ACGIH	American Conference of Governmental Industrial Hygienists
AMP	Alveolar Macrophage Pneumonia
AO	Adverse Outcome
AOP	Adverse Outcome Pathway
BALF	Bronchoalveolar Lavage Fluid
BIP	Bronchiolocentric Interstitial Pneumonia
CWP	Coal Worker’s Pneumoconiosis
DAD	Diffuse Alveolar Damage
DM	Dust Macule
ECHA	European Chemicals Agency
ERS/ATS	European Respiratory Society/American Thoracic Society
fPDF	Fibrotic Pulmonary Dust Foci
GIP	Giant Cell Interstitial Pneumonia
IARC	International Agency for Research on Cancer
IE	Initiating Event
ILO	International Labor Organization
IPF	Idiopathic Pulmonary Fibrosis
JOHAS	Japan Organization of Occupational Health and Safety
KE	Key Event
LDH	Lactate Dehydrogenase
MDF	Mixed Dust Fibrosis
NAMs	New Approach Methodologies
NIOSH	National Institute for Occupational Safety and Health
NSIP	Non-Specific Interstitial Pneumonia
OECD	Organization for Economic Co-operation and Development
OEL	Occupational Exposure Limit
OSHA	Occupational Safety and Health Administration
PDF	Pulmonary Dust Foci
PMF	Progressive Massive Fibrosis
PSLTs	Poorly Soluble Low Toxicity Particles
PSPs	Poorly Soluble Particles
ROS	Reactive Oxygen Species
TiO_2_	Titanium Dioxide
TiO_2_ NPs	Titanium Dioxide Nanoparticles
TLV	Threshold Limit Value
UIP	Usual Interstitial Pneumonia
